# Novel risk factors associated with retained placenta after vaginal birth

**DOI:** 10.1002/ijgo.15978

**Published:** 2024-10-19

**Authors:** Basel H. Nasser, Jimmy E. Jadaon, Nibal Awad‐Khamaisy, Luna Abo Lfoul, Israel Hendler

**Affiliations:** ^1^ Department of Obstetrics and Gynecology, Nazareth Hospital, Affiliated with The Azrieli Faculty of Medicine Bar‐Ilan University Nazareth Israel; ^2^ Bar‐Ilan University The Azrieli Faculty of Medicine Israel

**Keywords:** novel risk factors, obstetrics, perinatal care, postpartum period, retained placenta

## Abstract

**Objective:**

To evaluate maternal and obstetric risk factors associated with retained placenta following singleton live vaginal births.

**Methods:**

We conducted a retrospective cohort study of women diagnosed with retained placenta after singleton live vaginal birth at or after 24 weeks of gestation, compared in a 1:2 ratio with women who had uncomplicated vaginal deliveries. The study and control groups were matched for maternal age, gestational age, and parity. Multivariate regression analysis assessed potential risk factors related to retained placenta.

**Results:**

In all, 15,260 women who delivered at our medical center(both vaginal and non‐vaginal)between 2015 and 2022, 170 (1.1%) were diagnosed with retained placenta. Ninety‐nine women (0.65%) who met the inclusion criteria were matched with 198 controls (1.3%). Multivariate logistic regression identified potential risk factors not previously described for retained placenta, including in vitro fertilization (OR 3.8, 95% CI 1.3–11.7, *P* = 00.018), large‐for‐gestational‐age fetuses (OR 28.2, 95% CI 5.4–148.5, *P* = 00.029), and endometriosis (OR 8.2, 95% CI 0.92–20, *P* = 00.024). Additional risk factors included pre‐eclampsia, labor induction, vacuum‐assisted delivery, and prolonged second‐stage labor.

**Conclusion:**

This study identifies critical risk factors for retained placenta, highlighting the importance of early identification to improve maternal and neonatal outcomes.

## Synopsis

Our study highlights unreported risk factors associated with retained placenta, such as macrosomia, in vitro fertilization, and endometriosis.

## 1 INTRODUCTION

Retained placenta is the second leading cause of postpartum hemorrhage. The term “retained placenta” refers to the failure of the placenta to spontaneously expel within 30 min following vaginal birth. This definition is particularly applicable in the third trimester, when the third stage of labor involves active management, such as the administration of a uterotonic agent before placenta delivery and controlled cord traction. With this managed setting, approximately 98% of placentas are expelled within the specified timeframe.^1^, ^2^ Known risk factors for retained placenta include a history of retained placenta, preterm delivery, previous uterine surgery, previous pregnancy termination, miscarriage or curettage, grand multiparity (more than five previous deliveries), and congenital uterine anomalies.^3^ Data concerning other possible risk factors for retained placenta are scarce. The present study aimed to determine the incidence of and identify maternal, pregnancy, and labor characteristics associated with retained placenta in women after spontaneous vaginal birth without previous cesarean section or intrauterine procedures.

## 2 MATERIALS AND METHODS

A retrospective case–control cohort, of women who had retained placenta after singleton live vaginal delivery at or after 24 weeks of pregnancy with vertex presentation managed by manual removal of the placenta, between January 1, 2015, and December 31, 2022, compared with women who had a normal vaginal delivery without complications. The control group was matched in a 1:2 ratio for maternal age, gestational age, and parity. The researcher also accounted for previous studies highlighting common issues related to retained placenta. However, the inclusion criteria were specifically limited to pregnant women over the age of 18 years. This method is susceptible to selection bias because of the patient‐record inclusion process. Individuals with retained placenta who are in poorer health tend to have more comprehensive data available compared with healthier patients or instances where one patient group is overrepresented in the sample. As a result, the composition of study participants may be biased, as it may not accurately reflect the general population's distribution of risk factors associated with retained placenta after vaginal delivery. We used systematic matching for the control group, identifying women from a hospital delivery list, matched on maternal age, gestational age, and parity, without selection bias. Although propensity score matching was not used, we believe that the demographic variables controlled for were sufficient for the study.

Inclusion criteria were: pregnant women delivered between 24 and 42 weeks of pregnancy, singleton pregnancies, women aged 18 years or older, and vertex presentation.

Previous cesarean section, other uterine surgeries, dilatation and curettage; gestational age less than 24 weeks; intrauterine fetal demise; known uterine anomalies; twin pregnancies; and non‐vertex presentations were all considered exclusion criteria.

We excluded previous uterine surgeries to ensure that the observed associations with retained placenta were not confounded by surgical alterations to the uterus, which are well‐established risk factors.

Given the potential for selection bias in the study criteria, a systematic approach was employed to minimize its influence. The women in the control group were selected using a structured process, ensuring no selection bias in the method. The control group was formed as follows: a list of women who gave birth at our hospital between 2015 and 2022 and met the inclusion criteria (15 260 women) was prepared. Those meeting the exclusion criteria were removed from the list. Women whose placenta did not separate were marked as “case” (99 women). The list was then sorted by birth date, and for each “case,” two women with the same age, gestational age, and parity who gave birth near the same date were selected for the control group (198 women). To further reduce bias, the groups were matched on parity. Although propensity score matching could be advantageous in some studies, the researcher determined that matching on key demographic variables was sufficient for this analysis. This matching method provided a robust control group for the present study. Data were collected from patients' computerized medical records. Demographic and medical characteristics included; maternal age, body mass index (calculated as weight in kilograms divided by the square of height in meters), smoking habits, gravidity and parity, chronic hypertension, thrombophilia, systemic lupus erythematosus (SLE), previous retained placenta, endometriosis, pregestational diabetes, thyroid disease, and cases involving in vitro fertilization (IVF).

Obstetrical and delivery characteristics included gestational age, premature rupture of membranes, labor induction, duration of the second stage, type of analgesia, mode of delivery (normal vaginal delivery versus instrumental delivery), pre‐eclampsia, estimated maternal blood loss, neonatal weight, postpartum hemorrhage, intrapartum fever, and endometritis.

At our medical institution we routinely perform active management of the third stage of labor: administration of uterotonic agents immediately postpartum; 10 units of in vitro oxytocin diluted in 1 L of normal saline at a rate of 1–2 mL/min as well as controlled cord traction for expulsion of the placenta. Retained placenta was defined as a placenta that failed to separate for more than 30 min after fetal delivery and was manually removed. Delayed cord clamping is a recognized practice for managing the third stage of labor, but research has not yet established a direct connection between its use and the incidence of retained placenta. Further studies are needed to determine if any relationship exists, particularly considering variations in clamping timing and potential interactions with other management strategies.

All characteristics were compared between deliveries with the retained placenta (study group) and the control group.

Our primary goal was to evaluate the risk factors (maternal and obstetrical characteristics) associated with retained placenta without a history of uterine procedures.

Our secondary goal was to assess maternal outcomes and delivery complications, including the incidence of postpartum hemorrhage, endometritis, the need for blood transfusion, incidence of hypovolemic shock, prolonged hospital stay (>4 days), and intrapartum fever greater than 38°C.

The study was approved by the Medical Center Nazareth Hospital EMMS Ethics Committee in October 2022 (approval number 50‐22‐EMMS).

We used the *χ* ^2^ test or the Fisher exact test to examine the relationships between risk factors and the dichotomous outcome variable (retained placenta versus spontaneous placental expulsion). The relation between the dichotomous outcome variable (retained placenta versus spontaneous placental expulsion) and the normally distributed variables was examined using the *t* test.

The relation between the dichotomous outcome variable (retained placenta versus spontaneous placental expulsion) and the non‐normally distributed variables was examined using the Mann–Whitney (Wilcoxon) *U* test.

We used logistic regression models to examine the multivariate relationships between risk factors and the odds for retained placenta. Before introducing the variables into the model, the multicollinearity of the variables was analyzed, using the Variance Inflation Factor statistic. The statistical analyses were performed using a statistical software package for data analysis (SAS OnDemand for Academics, version 3.8, Enterprise Edition). A *P* value of 0.05 or less was considered statistically significant.

Clarification of the statistical methods is essential for transparency and reproducibility. Recognizing this need, the researchers employed several methods. To evaluate the relationships between risk factors and the dichotomous outcome variable (retained placenta versus spontaneous placental expulsion), both univariate and multivariate analyses were conducted. For categorical variables, the *χ* ^2^ test or Fisher exact test, as appropriate, was used to examine associations with the outcome.

For continuous variables, the researchers first assessed their distribution through visual inspection, specifically examining histograms. Based on this assessment, normally distributed variables were analyzed using the *t* test, while non‐normally distributed variables were evaluated with the Mann–Whitney *U* test. To explore multivariate relationships between risk factors and the odds of retained placenta, logistic regression modeling was employed.

Variables identified as significant in the univariate analysis were included in the logistic regression model. Before inclusion, multicollinearity among the independent variables was assessed using the Variance Inflation Factor. All statistical analyses were conducted using the SAS OnDemand for Academics software package (version 3.8, Enterprise Edition). A *P* value of 0.05 or less was considered statistically significant.

The normality of the variables was evaluated through visual inspections, including histograms. Normally distributed variables were analyzed with the *t* test, while non‐normally distributed variables were tested using the Mann–Whitney *U* test. Multivariate logistic regression was used to assess the relationship between risk factors and retained placenta, with a Variance Inflation Factor used to assess multicollinearity. Additionally, we examined the influence of analgesia (epidural, sedation, or none) on the risk of retained placenta while accounting for other factors such as intervention type (e.g. labor induction) and the duration of labor.

## 3 RESULTS

During the study period, 15 260 women underwent delivery at our medical center, 170 (1.1%) were diagnosed with retained placenta.

Ninety‐nine women (0.65%) met the inclusion criteria for the retained placenta group, they were matched with 198 (1.3%) controls. Power calculations were conducted using SAS software (SAS OnDemand for Academics, version 3.8). For example, in the present study, 15% (*n* = 22) of women with IVF pregnancies experienced retained placenta, compared with 85% (*n* = 275) of women with spontaneous pregnancies. These calculations confirmed that the sample size was adequate to reject the null hypothesis, supporting the alternative hypothesis that women with spontaneous pregnancies had a lower rate of retained placenta at a significance level of 0.05.

### 3.1 Demographic characteristics and chronic diseases

As planned the groups were matched for maternal age, parity, and gestational age at birth (Table 1).

**TABLE 1:** Demographic and chronic disease characteristics for the study and control groups.^a^

Characteristics	Spontaneous placental expulsion (*n* = 198)	Retained placenta (*n* = 99)	*P* value
Age, years	32 (29–37)	32 (29–37)	>0.999
Parity	2 (1–3)	2 (1–3)	>0.999
Gestational age at delivery, weeks	38.89 ± 2.17	38.9 ± 2.17	>0.999
Preterm delivery <37 weeks	12 (6%)	6 (6%)	>0.999
BMI >25	37 (19%)	29 (29%)	0.038
Chronic hypertension	15 (8%)	16 (16%)	0.022
Smoking	27 (14%)	18 (18%)	0.3031
IVF	7 (4%)	15 (15%)	<0.001
Thrombophilia^b^	11 (6%)	6 (6%)	0.859
SLE	1 (1%)	4 (4%)	0.025
Pregestational diabetes	17 (9%)	12 (12%)	0.333
Thyroid disease	10 (5%)	11 (11%)	0.054
Previous retained placenta	0 (0%)	8 (8%)	<0.001
Endometriosis^c^	0 (0%)	4 (4%)	0.004

Abbreviations: BMI, body mass index (calculated as weight in kilograms divided by the square of the height in meters); IVF, in vitro fertilization; SLE, systemic lupus erythematosus.

^a^ Data are presented as mean ± standard deviation for variables with a normal distribution, as median (interquartile range) for variables with an abnormal distribution, or as number (percentage) as appropriate.

^b^ Thrombophilia including: Factor V Leiden, prothrombin G20210A gene mutation, deficiencies in protein C and protein S, as well as antithrombin and acquired thrombophilia antiphospholipid syndrome.

^c^ Endometriosis, the presence of which was confirmed by biopsy.

The following characteristics were more prevalent in the retained placenta group compared with the control group: women with body mass index greater than 25 (29% versus 19%, *P* = 0.038), chronic hypertension (16% versus 8%, *P* = 0.022), IVF (15% versus 4%, *P* < 0.001). SLE (4% versus1%, *P* = 0.025). Previous retained placenta (8% versus 0%, *P* < 0.001), and endometriosis (4% versus 0%, *P* = 0.004) respectively (Table 1).

### 3.2 Obstetrical and delivery characteristics

A higher percentage of women with retained placenta underwent labor induction (96% versus 66%, *P* < 0.001), mainly with prostaglandin E2 (31% versus 12%, *P* < 0.001). Premature rupture of membranes (27% versus 15%, *P* = 0.012), and pre‐eclampsia (8% versus 2%, *P* = 0.012). Epidural analgesia was more frequent in women with a retained placenta (86% versus 26%, *P* < 0.001), as well as a longer second stage (>3 h) (47% versus 5%, *P* < 0.001) and more vacuum‐assisted deliveries (16% versus 2%, *P* < 0.001). Birth weight less than 2500 g or more than 4000 g was more prevalent in women with retained placenta (7% versus 2%, *P* < 0.001 and 15% versus 2% *P* < 0.001, respectively) (Table 2).

**TABLE 2:** Obstetrics‐related characteristics of women with retained and non‐retained placenta.^a^

Characteristics	Spontaneous placental expulsion (*n* = 198)	Retained placenta (*n* = 99)	*P* value
Labor induction	130 (66%)	95 (96%)	<0.001
Prostaglandin E_2_	24 (12%)	31 (31%)	<0.001
Double‐balloon catheter	4 (2%)	6 (6%)	0.068
Oxytocin^b^	102 (52%)	58 (59%)	0.249
Duration of second stage >3 h	10 (5%)	47 (47%)	<0.001
PROM	30 (15%)	27 (27%)	0.012
Type of anesthesia			
Epidural	52 (26%)	85 (86%)	<0.001
Sedation	67 (34%)	11 (11%)	
None	79 (40%)	3 (3%)	
Type of delivery			
Spontaneous	195 (98%)	82 (84%)	<0.001
Vacuum	3 (2%)	16 (16%)	
Fetal weight, g			
<2500	4 (2%)	7 (7%)	<0.001
2500–4000	191 (96%)	77 (78%)	
>4000	2 (2%)	15 (15%)	
Pre‐eclampsia	4 (2%)	8 (8%)	0.012

Abbreviation: PROM, premature rupture of membrane.

^a^ Data are presented as numbers (percentage).

^b^ Oxytocin administration includes oxytocin for induction and/or augmentation of delivery.

### 3.3 Maternal and delivery outcomes associated with retained placenta

Women with retained placenta were more likely to experience postpartum hemorrhage (36% versus 13%, *P* < 0.001) and to receive blood products (13% versus 2%, *P* < 0.001), respectively. Additionally, they had a higher rate of endometritis and intrapartum fever (14% versus 0%, *P* < 0.001 and 8% versus 3%, *P* = 0.005) and prolonged hospital stay of 4 days or longer (41% versus 7%, *P* < 0.001) (Table 3).

**TABLE 3:** Maternal outcome and delivery complications.^a^

Outcome/complication	Spontaneous placental expulsion (*n* = 198)	Retained placenta (*n* = 99)	*P* value
PPH	26 (13%)	36 (36%)	<0.001
Blood transfusion	6 (3%)	36 (36%)	<0.001
Hypovolemic shock^b^	4 (2%)	13 (13%)	<0.001
Endometritis^c^	0 (0%)	14 (14%)	<0.001
Prolonged hospital stay (≥4 days)	13 (7%)	41 (41%)	<0.001

Abbreviation: PPH, postpartum hemorrhage (maternal blood loss >500 mL after vaginal delivery).

^a^ Data are presented as numbers (percentage);

^b^ Hypovolemic shock: blood loss resulting in a blood pressure of <90/60 associated with hemodynamic instability.

^c^ Endometritis: fever >38°C within 24 h post‐delivery associated with lower abdominal pain and uterine tenderness.

### 3.4 Multivariate logistic regression analysis

Multivariable logistic regression analysis (Table 4) revealed that the following characteristics were independently associated with increased risk of a retained placent—IVF pregnancy (odds ratio [OR] 3.8, 95% confidence interval [CI] 1.3–11.7, *P* = 0.018), labor induction (OR 21.8, 95% CI 5.5–86.8, *P* < 0.001), pre‐eclampsia (OR 4.5, 95% CI 1.1–17.5, *P* = 0.031), duration of the second stage greater than 3 hours (OR 3.9, 95% CI 1–15.1, *P* < 0.001), instrumental delivery (vacuum versus vaginal delivery) (OR 2.3, 95% CI 1.2–4.5, *P* = 0.010), small for gestational age (small versus appropriate for gestational age) (OR 16.8, 95% CI 2.7–103.7, *P* = 0.223), large for gestational age (large versus appropriate for gestational age) (OR 28.2, 95% CI 5.4–148.5, *P =* 0.029).

**TABLE 4:** Multivariable logistic regression analysis—factors associated with retained placenta.^a^

Risk factor	aOR	95% CI	*P* value
Pre‐eclampsia	4.5	1.1–17.5	0.031
Duration of second stage, h (>3 vs. <2)	3.9	1.0–15.1	<0.001
Labor induction	21.8	5.5–86.8	<0.001
Endometriosis	8.2	0.92–20	0.024
IVF	3.8	1.3–11.7	0.018
Fetal weight^b^			
SGA vs. AGA	16.8	2.7–103.7	0.223
LGA vs. AGA	28.2	5.4–148.5	0.029
Type of delivery (vacuum vs. vaginal delivery)	2.3	1.2–4.5	0.010

Abbreviations: AGA, appropriate for gestational age; aOR, adjusted odds ratio; CI, confidence interval; IVF, in vitro fertilization; LGA, large for gestational age; SGA, small for gestational age; SLE, systemic lupus erythematosus.

^a^ Factors included in the multivariable regression model were independent variables found significantly different between groups in the univariate analysis.

^b^ Fetal weight: SGA indicates birth weight below the 10th centile; LGA indicates birth weight above the 90th centile.

## 4 DISCUSSION

The present study investigated and aimed to identify previously unreported risk factors associated with retained placenta, focusing on women who had undergone vaginal births with no previous intrauterine intervention. Over 8 years, the incidence of retained placenta was 1.1%, which was consistent with previously reported rates of 0.5%–3%.^4^, ^5^, ^6^ Retained placenta was associated with several risk factors, some of which have not been previously described, such as macrosomia, IVF, and endometriosis, as well as reported risk factors such as pre‐eclampsia, labor induction, regional anesthesia, instrumental delivery, and prolonged second stage of labor. In our study cohort, women undergoing assisted reproductive technology independently contributed to an elevated risk of retained placenta with an OR of 3.8 compared with women who conceived naturally. The identified risk factors, such as IVF, macrosomia, and labor induction, may influence the physiologic processes of placental separation through hormonal or mechanical influences, as supported by previous studies on abnormal placental adherence and vascular stress. These findings underscore the multifactorial nature of retained placenta, necessitating a deeper exploration of the underlying pathophysiology.

Aziz et al.^7^ investigated the relationship between IVF and the length of the third stage. The authors concluded that cryopreserved embryo transfer (donated or autologous) without controlled ovarian hyperstimulation was not associated with a longer third stage but significantly increased the risk for manual removal of the placenta.

Our finding of an increased risk of retained placenta associated with IVF pregnancies is consistent with previous research, such as the study by Wertheimer et al.,^8^ who found that complications of the third stage of labor were more prevalent in IVF pregnancies. These findings suggest that IVF may contribute to a higher risk of retained placenta.

We noted a significantly higher risk of retained placenta in women with endometriosis.^9^ This group of women is also known to have an increased risk for placenta previa and excessive bleeding during cesarean section and systematic literature review supports our interpretation of the identified retained placenta risk factors, encompassing endometriosis and assisted reproductive technologies.^10^

Endometriosis has been associated with an increased risk of various obstetric complications, as highlighted by Kobayashi et al.^11^ In their review of the relationship between endometriosis and obstetric complications, the authors emphasized that women with endometriosis are more likely to experience retained placenta. This complication may stem from the pathophysiologic changes associated with the condition.

One of the proposed mechanisms is the alteration of uterine peristalsis in women suffering from endometriosis, which may impede the normal migration of the blastocyst during implantation. Such disturbances can lead to the improper placement of the blastocyst and consequently elevate the risk of conditions like placenta previa, and structural and functional modifications within the inner layer of the myometrium, particularly in the junctional zone, can hinder the physiologic remodeling of the spiral arteries in the uteroplacental bed. This failure is crucial as it can adversely affect placentation and is frequently observed in cases of retained placenta, affirming the association between defective placentation and the prevalence of retained placenta in patients with endometriosis.

Furthermore, a multicenter retrospective study highlighted that assisted reproductive technologies significantly increased the risk of retained placenta, necessitating manual removal of the placenta and leading to postpartum hemorrhage.^12^ Additionally, women with retained placenta were more likely to experience premature rupture of membranes, and large‐for‐gestational‐age infants.^13^ Macrosomia had a strong association with a retained placenta (OR 28.2) compared with infants classified as appropriate for gestational age.

We hypothesize that mechanical factors associated with macrosomic infants, such as increased shoulder width and head circumference, can hinder the effective contraction of the uterus and impair the natural separation of the placenta. This may lead to incomplete placental expulsion, triggering complications like uterine atony and postpartum hemorrhage. The intricate interplay of biomechanical and physiologic factors underscores the importance of vigilant obstetric management in cases of macrosomia to minimize the likelihood of retained placenta and its associated adverse outcomes.

Placenta‐associated pregnancy complications such as chronic hypertension and pre‐eclampsia lead to hypoperfusion and placental oxidative stress.^14^ This association extends to other maternal characteristics linked to abnormal placentation, specifically SLE.^15^ Individuals with SLE encounter an increased risk of adverse pregnancy outcomes, mainly attributable to impaired placentation. These changes predominantly encompass abnormalities in placental vascularity and coagulation, ultimately resulting in impaired trophoblastic invasion.^16^

In the present study, women with pre‐eclampsia had 4.5 times higher odds of experiencing retained placenta compared with those without pre‐eclampsia.

Women who underwent labor induction had a 21.8 times higher likelihood of experiencing retained placenta compared with those who did not undergo induction(mainly the use of prostaglandins). Their use can increase the risk of retained placenta for several reasons. First, if uterine contractions are excessively stimulated, they can lead to uterine atony—a condition where the uterus lacks adequate muscle tone to contract effectively after delivery. This diminished contractile ability may prevent the complete expulsion of the placenta.

Women who had an instrumental delivery using vacuum extraction had a 2.3 times higher likelihood of experiencing retained placenta compared with those who had a vaginal delivery.

We identified an association between retained placenta and regional analgesia. Upon further review, this association may be confounded by the longer duration of labor and higher rates of labor induction seen in the analgesia group, both of which are independent risk factors for retained placenta. Therefore, it is difficult to conclude that analgesia alone increases the risk of retained placenta. Further studies are required to disentangle the effects of analgesia from these other interventions. Although some studies suggest a possible link between epidural analgesia and retained placenta, the mechanism remains unclear. Epidural analgesia might depress the autonomic nervous system, potentially affecting uterine contractions and increasing the risk of incomplete placental expulsion. It is important to note that pethidine (meperidine hydrochloride) for labor analgesia did not show a similar association with retained placenta.^17^

Obstetrical complications and intrapartum conditions are associated with placental disease, particularly maternal vascular hypoperfusion. This leads to inadequate contraction of the retroplacental uterine wall, affecting placental detachment during the third stage of labor.^18^ Importantly, in our study, women with a prolonged second stage of labor lasting more than 3 h had a 3.9 times higher likelihood of experiencing retained placenta compared with those with a shorter second stage, emphasizing that labor dystocia in the second stage increases the likelihood of subsequent retained placenta.

In the present study, a history of retained placenta appears to be linked to an increased likelihood of recurrence in subsequent vaginal deliveries. These findings align with existing literature. Specifically, women with a history of retained placenta during vaginal delivery exhibited a significantly heightened risk of recurrence in subsequent deliveries. Notably, a study involving over 280 women in Denmark reported a substantial increase in the risk of recurrence, reaching approximately 25%.^19^

Contrary to the findings of Romero et al.,^20^ the present study did not reveal a higher incidence of retained placenta in preterm deliveries compared with term parturients. This could be because of the low incidence of preterm birth, in our population accounting for only 6% of the total births. This limited number of preterm births may have contributed to the lack of association between retained placenta and preterm birth. The present study did not identify any significant association between the retained placenta and maternal age, smoking, pregestational diabetes, or thrombophilia. These factors may not be strong predictors or contributors to the development of retained placenta in our study population. Future research should focus on the external validation of our findings in other populations and healthcare settings. Additionally, further studies are needed to explore the applicability of our identified risk factors across different geographic, socioeconomic, and clinical environments. This would help confirm the robustness of our results and provide more comprehensive guidelines for the management of retained placenta.

Our cohort study demonstrates notable strengths as a large retrospective investigation performed in a single medical center with consistent obstetrical protocols during an 8‐year period. The utilization of 2:1 matched controls enhances the reliability of the study. The capacity to extrapolate demographics, obstetrical history, and chronic diseases not previously studied enriches the research. Nevertheless, the retrospective design and potential data limitations, including the absence of certain data points and histopathologic findings, introduce inherent limitations.

Confidence intervals are crucial when considering study limitations, as they highlight the uncertainty of the estimates. Although the study's sample size was sufficient for the analysis, wide confidence intervals in some variables, such as IVF pregnancies, suggest potential variability that may affect the precision of the estimates. Future studies with larger populations could further refine these findings.

Some of the estimates of ORs in the multivariate model are indeed broad, but as the ORs are similar to the ORs in the univariate models, we think they reflect reality. Further studies are needed to prove these hypotheses.

It is important to note that the discussions in the present study may not fully align with its primary purpose. Despite the correlations observed, they may be less strongly associated with retained placenta than initially suggested. Consequently, the study may fall short of providing cohesive and coherent insights into the pathophysiologic mechanisms needed to support its findings. Future studies could benefit from developing a nomogram to better predict the risk of retained placenta based on the identified risk factors.

In conclusion, the present study highlights the importance of early identification of previously unreported risk factors such as macrosomia, IVF, and endometriosis, while the study provides significant insights, some areas warrant further investigation. Future research could focus on elucidating the specific mechanisms by which macrosomia, IVF, and endometriosis contribute to the development of retained placenta.

Our findings underscore the need for informed patient counseling regarding potential complications like retained placenta and postpartum hemorrhage. Understanding the pathophysiology of these disorders is crucial for developing effective preventive and treatment strategies. Our novel findings offer promise for physicians in assessing these risks pre‐delivery, thus providing valuable insights into maternal care.

## AUTHOR CONTRIBUTIONS

B.H.N., I.H., N.A.‐K., L.A.L., and J.E.J. contributed to conception, acquisition, analysis, and interpretation of the data, and to drafting the manuscript. All authors agree with the final version of the manuscript and its submission to the *International Journal of Obstetrics and Gynecology*.

## CONFLICT OF INTEREST STATEMENT

The authors declare no conflict of interest.

## Data Availability

Research data are not shared.
